# The efficacy of alcelaphine herpesvirus-1 (AlHV-1) immunization with the adjuvants Emulsigen^®^ and the monomeric TLR5 ligand FliC in zebu cattle against AlHV-1 malignant catarrhal fever induced by experimental virus challenge

**DOI:** 10.1016/j.vetmic.2016.09.019

**Published:** 2016-11-15

**Authors:** Felix Lankester, Ahmed Lugelo, Dirk Werling, Nicholas Mnyambwa, Julius Keyyu, Rudovick Kazwala, Dawn Grant, Sarah Smith, Nevi Parameswaran, Sarah Cleaveland, George Russell, David Haig

**Affiliations:** a Boyd Orr Centre for Population and Ecosystem Health, Institute of Biodiversity, Animal Health & Comparative Medicine, University of Glasgow, Glasgow, UK; b Paul G. Allen School for Global Animal Health, Washington State University, Pullman, WA 99164, USA; c School of Life Sciences and Bioengineering, Nelson Mandela African Institution of Science & Technology, Arusha, Tanzania; d Faculty of Veterinary Medicine, Sokoine University of Agriculture, Morogoro, Tanzania; e Royal Veterinary College, Department of Pathology and Pathogen Biology, London, UK; f Tanzanian Wildlife Research Institute, Arusha, Tanzania; g Moredun Research Institute, Midlothian, Edinburgh, UK; h School of Veterinary Medicine and Science, University of Nottingham, Nottingham, UK

**Keywords:** Malignant catarrhal fever, Alcelaphine herpesvirus-1, Vaccine trial, Wildebeest, Adjuvant, Bacterial flagellin

## Abstract

Malignant catarrhal fever (MCF) is a fatal disease of cattle that, in East Africa, follows contact with wildebeest excreting alcelaphine herpesvirus 1 (AlHV-1). Recently an attenuated vaccine (atAlHV-1) was tested under experimental challenge on Friesian-Holstein (FH) cattle and gave a vaccine efficacy (VE) of approximately 90%. However testing under field conditions on an East African breed, the shorthorn zebu cross (SZC), gave a VE of 56% suggesting that FH and SZC cattle may respond differently to the vaccine. To investigate, a challenge trial was carried out using SZC. Additionally three adjuvant combinations were tested: (i) Emulsigen^®^, (ii) bacterial flagellin (FliC) and (iii) Emulsigen^®^ + bacterial flagellin. We report 100% seroconversion in all immunized cattle. The group inoculated with atAlHV-1 + Emulsigen^®^ had significantly higher antibody titres than groups inoculated with FliC, the smallest number of animals that became infected and the fewest fatalities, suggesting this was the most effective combination. A larger study is required to more accurately determine the protective effect of this regime in SZC. There was an apparent inhibition of the antibody response in cattle inoculated with atAlHV-1 + FliC, suggesting FliC might induce an immune suppressive mechanism. The VE in SZC (50–60%) was less than that in FH (80–90%). We speculate that this might be due to increased risk of disease in vaccinated SZC (suggesting that the vaccine may be less effective at stimulating an appropriate immune response in this breed) and/or increased survival in unvaccinated SZC (suggesting that these cattle may have a degree of prior immunity against infection with AlHV-1).

## Highlights

• Vaccination induces a pharyngeal antibody response in shorthorn zebu cross (SZC).
• Direct challenge with the AlHV-1 virus is effective at inducing MCF in SZC.
• Attenuated AlHV–1 + Emulsigen^®^ vaccine efficacy in SZC calculated to be 50%.
• Bacterial flagellin is not a good adjuvant as inclusion reduced antibody response.
• We provide evidence that non-fatal AlHV-1 infections occur in SZC.

## 1 Introduction

Malignant catarrhal fever (MCF), an often-fatal disease with a worldwide distribution, is caused by several γ-herpesviruses and affects many species of even-toed ungulates including cattle, bison and deer (Russell et al., 2009). The disease ranges from the sporadic to epidemic and occurs following transmission from an unapparent carrier host to MCF-susceptible species. Defined by the reservoir species from which the causative virus arises, two major epidemiological forms of MCF exist, wildebeest-associated (WA-MCF) (Plowright et al., 1960) and sheep-associated (SA-MCF) MCF (Reid et al., 1984). The clinical presentation is similar in both forms with affected animals suffering fever, oral epithelial lesions, corneal opacities, ocular/nasal discharge and, frequently, death. The pathogenesis is poorly understood but appears to involve an auto-destructive pathology resulting in lymphoid hyperplasia and vasculitis in a range of tissues (Anderson et al., 2008).

WA-MCF occurs primarily in sub-Saharan Africa wherever wildebeest come into contact with cattle. The causative pathogen, alcelaphine herpesvirus-1 (AlHV-1), is excreted by wildebeest calves (*Connochaetes taurinus*) in the three months following the brief annual calving period. To avoid disease, pastoralists move their cattle from wildebeest calving grounds, often to more marginal land tens of km away, at a time of year when the cattle condition is most vulnerable. Consequently, the economic costs of MCF can be significant (Lankester et al., 2015). These costs could be alleviated with the development of an efficacious vaccine.

SA-MCF, caused by ovine herpes virus-2 (OvHV-2) (Hart et al., 2007), has a worldwide distribution and represents the more economically significant form. Because no *in-vitro* propagation system exists for OvHV-2, vaccine development has focused on AlHV-1, which can be cultured *in-vitro*. OvHV-2 is phylogenetically related to AlHV-1 with significant DNA sequence identity (Hart et al., 2007) and, although the potential for a vaccine based on AlHV-1 to provide cross-protective immunity against SA-MCF is likely to be slight (Gailbreath et al., 2010, Taus et al., 2015), an effective AlHV-1 vaccine may, in addition to providing benefits to livestock keepers living in proximity with wildebeest, provide a basis for the development of a protective vaccine against OvHV-2.

Vaccine development has focused on AlHV-1 and recent United Kingdom based trials in British Friesian-Holstein (FH) cattle have demonstrated that, following two inoculations of attenuated AlHV-1 (atAlHV-1) mixed with the adjuvant Emulsigen^®^ (MVP Technologies, 2012), an effective barrier of AlHV-1-neutralizing antibodies can be induced in the mucosa of the oro-nasal pharynx, the presumed site for natural infection by the MCF viruses (Haig et al., 2008, Russell et al., 2012). The vaccine strategy gave FH cattle approximately 90% protection against AlHV-1 challenge while the duration of protection was limited to around six months. However a subsequent field trial experiment (Lankester et al., 2016) indicated that in shorthorn zebu cross (SZC) cattle the level of protection was only 56% under conditions of natural transmission. One explanation for the difference in the levels of protection seen is that FH and SZC cattle may respond differently to the vaccine. To begin investigating whether such a difference exists, a challenge trial equivalent to those performed in the UK was repeated on SZC cattle. The outcome of this trial is reported here.

This challenge trial was also used to investigate whether the efficacy of the vaccine could be improved by exploiting the adjuvant properties of particular ligands/agonists for toll-like receptors (TLR) on immune system cells. TLRs, which form part of the innate immune system and provide a first line of defense against infection, recognize pathogen-associated molecular pattern molecules (PAMPs) expressed by pathogens but not by mammalian hosts (Akira and Takeda, 2004, O’Neill et al., 2013). Up to thirteen TLRs are found in mammals, which recognize microbial and parasitic components such as unmethylated-CpG DNA (recognized by TLR9) and bacterial flagellin (recognized by TLR5). Importantly, engaging TLRs on antigen-presenting cells can generate inflammatory signals that influence the magnitude and type of the adaptive immune response that ensues. For this reason, TLR ligands have been the subject of much recent research into new generation adjuvants. For example the recombinant bacterial flagellin monomer (FliC), that is recognized by TLR5, has demonstrated adjuvant properties for antibody- and cell-mediated responses in several mammalian and avian species, including mucosal immune responses (Lee et al., 2015, Taylor et al., 2012). Therefore we aimed to compare the efficacy of the AlHV-1 vaccine when combined with one of three different adjuvant combinations: i) Emulsigen^®^ alone; ii) FliC alone; or iii) both adjuvants in combination.

## 2 Materials and methods

### 2.1 Ethical approval

The research was carried out with the approval of the Institutional Animal Care and Use Committee (IACUC) of the Tanzanian Wildlife Research Institute (TAWIRI), the Commission for Science and Technology (COSTECH, Tanzania) and the Tanzania Food and Drug Administration (permit nos. 2011-213-ER-2005-141 and 2012-318-ER-2005-141). All animal experiments were approved by, and were carried out in strict accordance with, the University of Nottingham and the Moredun Research Institute’s experiments and ethics committees and complied with the Home Office of Great Britain and Northern Ireland’s Animals (Scientific Procedures) Act 1986 under project license PPL 60/3839. To minimize suffering, the severity of MCF was not to exceed moderate (in the progression mild to moderate to severe) as determined by a clinical scoring system (Russell et al., 2012). As soon as cattle were determined to have moderate clinical signs they were euthanized.

### 2.2 Assessment of FliC efficacy in vitro

To assess the functional response of bovine TLR5 to ligand stimulation with FliC, the wild type bovine toll-like receptor-5 (boTLR5) was expressed as a protein fusion to Yellow Fluorescent Protein (YFP) in the plasmid vector pcDNA3-YFP (Metcalfe et al., 2014). The pcDNA3-YFP-boTLR5 and control pcDNA3-YFP plasmids were transfected separately into human embryonic kidney cells (HEK293) using a nucleofector kit (Annexa Biosystems, UK). Selective pressure was applied by addition of geneticin (G418, Invitrogen, UK, 600 μg/ml) until stably transfected cell lines were generated (2–3 weeks). Bovine TLR5 expression was confirmed by RT-PCR for boTLR5 as described (Metcalfe et al., 2014), flow cytometry and fluorescence microscopy for YFP. HEK cells expressing human TLR5 in pcDNA3 (293-htlr5) were used as control (Invivogen, Toulouse, France).

For the FliC − TLR5 HEK assay, the TLR5+ HEK cells (human and bovine TLR5) and negative controls were seeded at 2 × 10^5^ cells per well in a 24-well plate in 2 ml of Dulbecco’s modified Eagle’s medium (DMEM) + 10% FCS and incubated overnight at (37 °C, 5% CO2 in humidified air). Each cell line was then stimulated with four concentrations of two preparations of recombinant FliC: i) *Salmonella typhimiurium* Flagellin FliC (Enzo Life Sciences, Exeter, UK) < 0.05 EU/μg endotoxin and ii) as a positive control, endotoxin-free FliC (tlrl-flic; Invivogen, Source Bioscience LifeSciences, UK). Cells were stimulated with each of the FliC preparations at 0.1, 0.3, 0.6 and 1 μg/ml and supernatants were collected 24 and 48 h post stimulation. All treatments were performed in duplicate. Supernatants (500 μl) were cleared by centrifugation and stored at −20 °C. The functional response of bovine and human TLR5 HEK cells, and control cells, to ligands was measured by their production of the chemokine CXCL8, using the Quantikine ELISA measuring human CXCL8 (R&D systems, Abingdon, UK), as described recently (Willcocks et al., 2013).

### 2.3 In vivo vaccine trial − animals and virus

Forty clinically healthy Tanzanian shorthorn zebu cross (SZC) cattle (31 males and 9 females) of approximately six months of age were purchased from livestock markets in the Simanjiro District in northern Tanzania. All cattle were immunized against the locally prevalent and often fatal lymphoproliferative cattle disease East Coast fever (ECF) (Homewood et al., 2006). The animals were also given a single treatment against endo- and ectoparasites using 1 ml/50 kg body weight ivermectin (Ivomec^®^, Merial Animal Health, Essex, UK) administered by a subcutaneous injection. Every other week thereafter the cattle were sprayed with the ectoparasiticide alpha-cypermethrin (Paranex^®^, Farmbase Ltd, Dar es salaam, Tanzania), administered at 100 mg/l. All cattle were fitted with ear tags for identification. The cattle were housed at night in a traditional Maasai boma (corral) and, during the day, were grazed on community pastureland in the village of Emboreet (latitude −3.952239, longitude 36.47537).

The strains of the AlHV-1 virus used for vaccination and challenge were as described previously (Haig et al., 2008, Russell et al., 2012). Briefly, the virulent AlHV-1 (C500) strain virus was collected from cultures of bovine turbinate (BT) cells infected with a cell suspension derived from pooled lymphoid tissue from rabbits infected with AlHV-1 C500 that had developed MCF. Infected BT cell cultures were passaged onto fresh BT cells by a 1:4 split four times at peak cytopathic effect (approximately weekly) after which virulent virus was harvested from culture supernatants and cells following three rounds of freeze-thaw treatment. Cell-free virus supernatant was stored at −80 °C in batches and representative aliquots of each batch were titrated to allow calculation of the appropriate challenge dose. Titration measured 50% tissue-culture-infectious dose (TCID_50_) as described previously (Haig et al., 2008, Russell et al., 2012). Pathogenic virus challenge in this experiment was by intranasal inoculation of 10 ml of virus suspension with titre approximately 10^4^ TCID_50_/ml. We were confident that this dose would provide a lethal dose in SZC as it represented 50 x the LD_50_ virus dose as determined on FH cattle (Haig et al., 2008)_._ The attenuated AlHV-1 C500 strain, passaged more than 1000 times, was used as the source of virus for immunization (Handley et al., 1995). This cell-free virus was obtained from BT cell culture supernatants, clarified by centrifugation and stored in batches at −80 °C. Representative aliquots of attenuated AlHV-1 (10^7^ TCID_50_ atAlHV-1) were titrated as described for virulent AlHV-1.

### 2.4 Study design

The trial took place between October 2012 and February 2013 at a time of the year when wildebeest were not calving and had yet to migrate out of the nearby Tarangire National Park. Natural exposure to AlHV-1 was therefore deemed unlikely during the course of the trial. The 40 cattle were randomly assigned to one of five experimental groups (*n* = 8 for each group). Each group was primed on day zero and boosted on day 28 with an intramuscular injection in the upper neck with a vaccine mixture as specified in Table 1. The group sizes were similar to those described in the two UK-based immunization experiments upon which this trial was based (Haig et al., 2008, Russell et al., 2012). On day 77 after the primary inoculation all animals were challenged with 10 ml of 10^4^ TCID_50_/ml of virulent AlHV-1 given intranasally. In accordance with the previous UK experimental trials, the endpoint of the trial was three months (90 days) after challenge to allow the development of MCF in unprotected cattle. As the trial was held with the cooperation of local Maasai villagers in an area of Tanzania where MCF was endemic we decided, in recognition of local sensitivity toward the unnecessary slaughter of cattle, that the local community would retain all cattle that were healthy at the endpoint of the trial.

### 2.5 Sample collection and clinical analyses

Blood was collected in EDTA Vacutainers^®^ (BD Diagnostics, New Jersey, USA) from all animals at the day of primary and booster inoculation, and every two weeks thereafter until the end of the trial on day 168 (24 weeks after primary inoculation). Nasal secretion samples were collected using a tampon (Lil-lets^®^, regular) inserted into one nostril for 10 min. Following removal, the tampon was squeezed inside the barrel of a 20 ml syringe and the extracted nasal secretion collected. Plasma and nasal secretion samples were frozen at −20 °C and, prior to being exported to the UK for serological analysis, were heat-treated at 56 °C for 30 min. Buffy coat blood cells, also extracted from the uncoagulated blood, were stored frozen prior to DNA extraction for PCR detection of viral DNA. Clinical signs were monitored on a daily basis with animals recorded as ‘healthy’ or, if there was evidence of fever, excessive ocular/nasal discharge or anorexia, as ‘sick’. Sick animals were scored using a clinical scoring matrix (Russell et al., 2012) that ensured euthanasia took place prior to the onset of severe clinical signs. While histopathology is recognized as the gold standard for MCF diagnosis, and a post-mortem examination was carried out on all animals that were euthanized due to onset of MCF, the tissue samples that were collected were lost in transit to the UK and could not be analysed. Infection status was therefore defined based on a combination of clinical signs, analysis of virus-specific antibody responses and PCR detection of AlHV-1 DNA.

### 2.6 Detection of AlHV-1 in blood

Viral DNA was extracted from the frozen buffy coat samples at the Nelson Mandela African Institution of Science and Technology (Tanzania) using the ZR Viral DNA Kit™ (Zymo Research Corporation, USA) according to the manufacturer’s instructions. Viral DNA was assayed by a sensitive, nested PCR as described previously (Russell et al., 2012) and validated for use in the UK and Tanzania laboratories. Briefly, following a first round of AlHV-1 specific PCR, performed using AHV-POL1 (5′-ggctcataatctatgctactccac-3′) and AHV-POL2 (5′-attctccacaaactgttttgt-3′) primers, a 2 μl aliquot was used for a second hemi-nested round of PCR performed using AHV-POL internal forward primer (5′-ccaaaatgaagaccatctta-3′), and the first-round POL2 as reverse primer. All PCR reactions were carried out using BIOTAQ DNA polymerase (Bioline, London, UK − used in Tanzania) or HotStarTaq Plus DNA polymerase (Qiagen, Hilden, Germany − used in UK). Thermal cycling conditions were optimized according to the polymerase used. Hemi-nested PCR products were analysed by electrophoresis on 1.8% agarose gel and visualized, photographed and documented using Bio-Rad Gel Doc™ EZ system. AlHV-1 infection status during the challenge period was classified as positive if PCR analyses were positive at any of the three time points assayed (post-challenge day 28, 56 or 86). Although the hemi-nested PCR used here did not distinguish between vaccine and challenge virus, previous studies have shown that AlHV-1 DNA was not detected in the blood of any cattle vaccinated with the attenuated virus, even up to nine months after vaccination (Russell et al., 2012). Therefore PCR positive samples from trial cattle were considered to provide evidence of infection with challenge AlHV-1.

### 2.7 Analysis of antibody responses by ELISA

1.6.1 To quantify the systemic (plasma) and nasal mucosa total AlHV-1-specific and neutralizing antibody responses, a previously described ELISA and virus neutralization test were used respectively (Russell et al., 2012). ELISA values (difference between means of positive and negative antigen wells for each sample dilution) were used to calculate a relative titre for each test sample, determined with respect to a standard curve of pooled MCF-positive plasma diluted 1/20 to 1/6000. ELISA titre values have been expressed as the reciprocal of the calculated end-point dilution (e.g. 20–6000). All samples were assayed twice using multiple dilutions. To reduce the likelihood that false positive titres were counted, any sample that gave a calculated titre of less than 20 (i.e. below the range of the standard curve) was not considered positive.

### 2.8 Definitions of clinical MCF cases

Histopathological analyses were not performed on the post-mortem tissues of those cattle that did die, nor on the tissues of cattle that were alive (and were not sacrificed) at the end of the trial. Consequently PCR, antibody responses and clinical signs were used to determine cases of MCF. Case definitions were based on cattle being classified as symptomatic and on the detection of AlHV-1 DNA by PCR. In addition, previous studies have shown that vaccinated cattle that subsequently succumbed to MCF exhibited a significantly increased AlHV-1 − specific antibody titre after challenge (Haig et al., 2008, Russell et al., 2012). We were therefore able to use the induction of, or increase in, AlHV-1 − specific antibody titre following virus challenge as an indication of AlHV-1 infection. The various combinations of diagnostic evidence were classified as follows:

• Not infected: Cattle remained asymptomatic, were PCR negative, survived and, if unvaccinated, had no antibody response following challenge.
• Fatal AlHV-1 infection: Cattle were PCR positive post-challenge, had clinical signs compatible with MCF and subsequently succumbed to disease.
• Non-fatal AlHV-1 infection: Cattle survived and were either PCR positive post-challenge or showed an induction or increase in antibody response following challenge.
• Possible AlHV-1 infection: Cattle had clinical signs indicative of MCF but no PCR or antibody evidence of infection.

These case definitions allowed further division of cattle into those for which there was evidence of infection, hereafter termed ‘infected’ (case definition II and III) and those for which there was none, hereafter termed ‘uninfected’ (case definition I). Cases classified as definition IV were termed ‘possibly infected’, based on clinical signs only.

### 2.9 Vaccine efficacy and comparisons with the other trials

A calculation of vaccine efficacy (for preventing infection) was performed for the atAlHV-1 + Emulsigen^®^ formulation (Group 1). The same calculations were also made for comparison using data from the Tanzania-based field trial (Lankester et al., 2016) and the UK-based trial (Russell et al., 2012) (hereafter termed Russell), both of which used Emulsigen^®^ as an adjuvant, and the UK-based trial that used Freund’s adjuvant (Haig et al., 2008) (hereafter termed Haig). The formula is shown in Supporting information 2.

### 2.10 Statistical analyses

All plots and statistical analyses were made using the R language for statistical computing (Team, 2013). A *t*-test was used to determine if there were differences in the response of HEK cells expressing TLR5 to FliC ligands. A linear regression model was used to compare i) the effect that vaccination grouping had on AlHV-1-specific antibody titre and ii) the AlHV-1-specific and neutralizing antibody titres of vaccinated cattle that were classified as ‘uninfected’ or ‘infected’. Fisher's Exact Test for Count Data was used to test the differences in the proportions of cattle in i) each group that were either ‘uninfected’ or ‘infected’ and ii) the relationship between prior exposure and survival in control group cattle. Vaccine efficacy calculations were made using standard formulae (Orenstein et al., 1985).

## 3 Results

### 3.1 FliC stimulates HEK cells transfected with bovine TLR5 in vitro

**Fig. 1:**
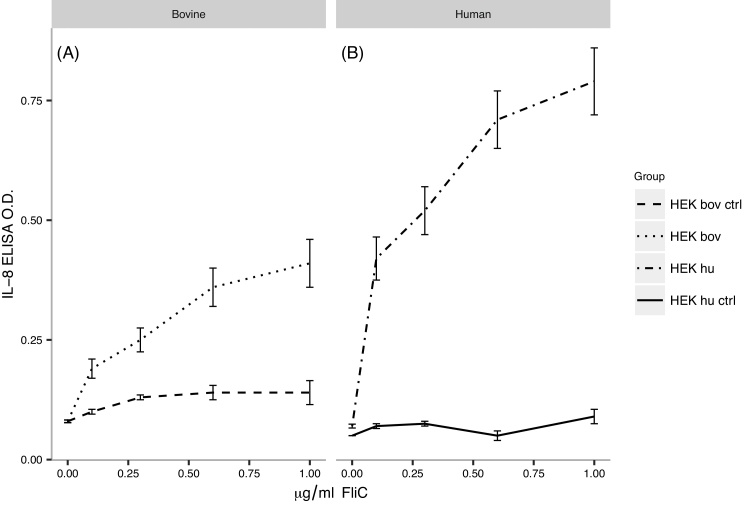
FliC efficacy assay: HEK bov (plot A) and HEK hu (plot B) are HEK cells expressing bovine and human TLR5 respectively. HEK bov ctrl and HEK hu ctrl are HEK cells containing control pcDNA3-YFP plasmid. The HEK bov and HEK hu cell responses to 0.1, 0.3, 0.6 and 1 μg/ml FliC were significantly different (*p* < 0.02). The HEK bov and the HEK hu cell responses to 0.1, 0.3, 0.6 and 1 μg/ml FliC were significantly different from their respective control cell responses (HEK bov ctrl; HEK hu ctrl) (*p* < 0.02 and *p* < 0.01 respectively).

In a first set of experiments, we assessed the response pattern of HEK cells expressing boTLR5 to different concentrations of FliC (Fig. 1). Similar to huTLR5, boTLR5 mediated a dose-dependent response to FliC, as shown by CXCL8 expression, although the response was significantly stronger using HEK cells expressing huTLR5 (*p* *<* 0.02). This is in line with recently published data on boTLR5 (Metcalfe et al., 2014). The response of HEK cells transfected with either bovine or human TLR5 to FliC was significantly higher than their respective control cell responses (*p* < 0.02 and *p* < 0.01 respectively for doses >0.1 μg/ml), which did not respond to either source of FliC. The positive control FliC (Invivogen) gave similar results to the adjuvant FliC in both cell types (not shown). Overall, these data confirmed that FliC could be potentially used as a vaccine adjuvant in the bovine system.

### 3.2 Emulsigen^®^, but not FliC supports the development of antibodies to AIHV-1

To establish baseline values of antibody titres, sera of all 40 animals were tested by ELISA and PCR at the start of the experiment (day 0). Thirty-eight animals tested negative, whereas two animals (901 and 926) exhibited low, but positive, AlHV-1-specific antibody titres. Four animals (914, 928, 936, 937) showed evidence of AlHV-1 DNA in blood mononuclear cells by PCR (Table 2 and Supporting information 1).

**Fig. 2:**
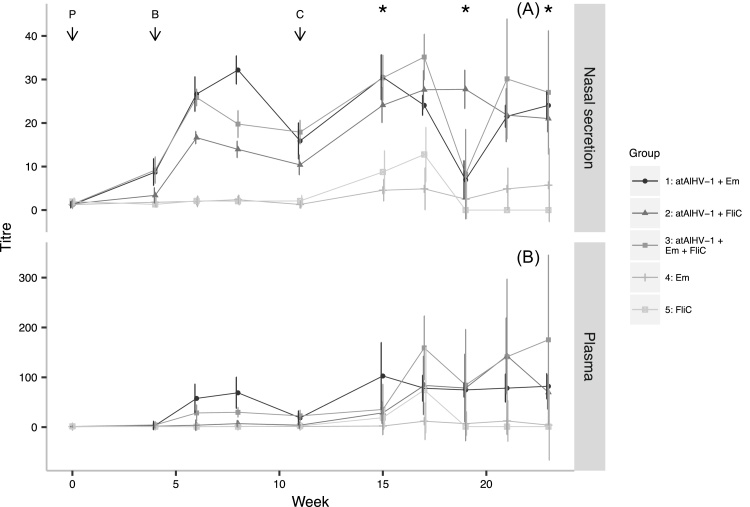
AlHV-1-specific antibody titres: The geometric mean nasal secretion (plot A) and plasma (plot B) total AlHV-1 − specific antibody titres (and 95% confidence intervals) for Groups 1–5 are shown for each sampling time point (atAlHV-1 = attenuated AlHV-1 virus, Em = Emulsigen^®^, FliC = flagellin monomer, P = primary vaccination, B = booster vaccination, C = virus challenge and * = PCR assay time-points). Group 1, atAlHV-1 + Emulsigen^®^; Group 2, atAlHV-1 + FliC; Group 3, atAlHV-1 + Emulsigen^®^ + FliC; Group 4, Emulsigen^®^; Group 5, FliC. The wide confidence intervals after virus challenge (C) indicate large individual variations (see text and Fig. 4).

Between boosting and challenge, all of the cattle in the atAlHV-1 vaccinated groups (1, 2 and 3) showed a rise in nasal secretion AlHV-1-specific antibody titres, whilst cattle in the atAlHV-1 vaccinated groups 1 and 3 showed a rise in plasma AlHV-1-specific antibody titres. Antibody titres peaked between weeks seven and eight, before declining again. In contrast, none of the cattle in either adjuvant control group (4 or 5) showed an AlHV-1-specific antibody response (Fig. 2). Group 1 had significantly higher geometric mean pre-challenge plasma titres than all other groups (*p* < 2 × 10^−16^, *t* = 10.4, df = 114), and significantly higher nasal secretion titres than all groups (*p* < 2 × 10^−16^, *t* = 16.4, df = 155) except Group 3. Furthermore, the plasma and nasal secretion titres of Group 2 were significantly lower than those of Groups 1 and 3 (*p* < 0.001, *t* = −6.1 (plasma) & *t* = −5.6 (NS)). Following virus challenge, geometric mean plasma and nasal secretion AlHV-1-specific antibodies increased in all three vaccinated groups, but error bars were large indicating a wide range of individual variation. Both control groups also showed a rise in virus-specific titre after virus challenge, but these were very low compared to the immunized groups (Fig. 2).

**Fig. 3:**
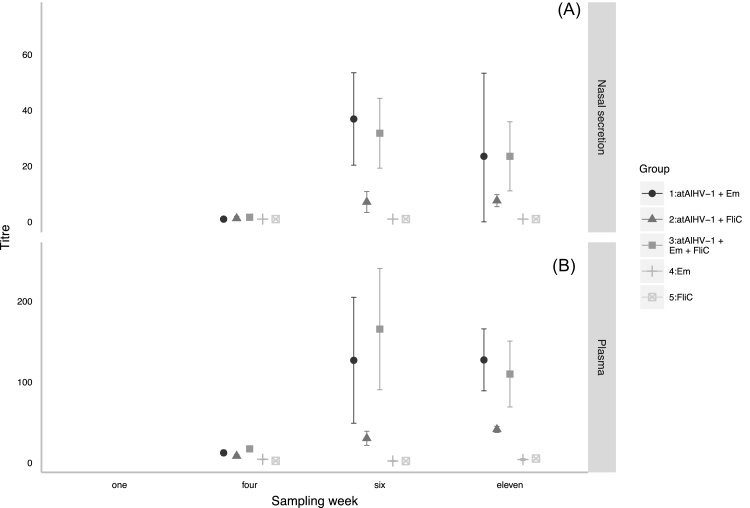
AlHV-1 – neutralizing antibody titres: The geometric mean AlHV-1 – neutralizing antibody titres (and 95% confidence intervals) in nasal secretion (plot A) and plasma (plot B) for each of Groups 1–5 are shown (atAlHV-1 = attenuated AlHV-1 virus, Em = Emulsigen^®^, FliC = flagellin monomer). Group 1, atAlHV-1 + Emulsigen^®^; Group 2, atAlHV-1 + FliC; Group 3, atAlHV-1 + Emulsigen^®^ + FliC; Group 4, Emulsigen^®^; Group 5, FliC. The primary inoculation occurred in week one, the booster in week four and the challenge in week eleven.

Regarding AlHV-1 − neutralizing antibodies, both control Groups 4 and 5 remained seronegative, whilst all of the animals in Groups 1, 2 and 3 seroconverted after the booster immunization (Fig. 3). Vaccinated Group 2 cattle had significantly lower geometric mean plasma and nasal secretion AlHV-1-neutralizing antibody titres than both Groups 1 and 3 *(p* < 0.001, *t* = −3.4, df = 74 (plasma); *p* < 0.001, *t* = −3.5, df = 73 (nasal secretion)).

Blood samples from all cattle were assayed for AlHV-1 DNA at three post-challenge occasions: week 15, 19 and 23. Where available, PCR was also performed on terminal blood samples taken from animals that succumbed to MCF. The results are shown in Supporting information 1. In Groups 1–5, AlHV-1 virus DNA was detected in two, four, six, five and six out of eight cattle, respectively. The post-challenge PCR status of each animal is summarized in Table 2.

The details of individual animal and treatment group survival or acquisition of MCF are shown in Table 2 and are summarized in Table 3.

### 3.3 Case descriptions

The majority of animals were classified as either case definition (CD) I (not infected) or CD II (fatal AlHV-1 infection), with a small number of animals (6) showing more complex combinations of signs (Table 2, Table 3).

In Group 1, two animals succumbed to MCF with clear clinical signs and detection of virus DNA in the blood post-challenge and were classified as CD II. Six animals survived with no clinical signs and no detection of AlHV-1 DNA in the blood at any time point after challenge. Of these, one animal (number 913), despite being asymptomatic and PCR negative, had a rising AlHV-1–specific antibody titre following challenge and was classified as CD III (non-fatal AlHV-1 infection). The other five were classified as CD I.

In Group 2, three animals survived with no clinical signs and no detection of virus DNA post-challenge and were classified as CD I. Four animals succumbed to MCF with clinical signs and virus DNA in the blood post-challenge and were classified as CD II. One animal (number 940) showed clinical signs indicative of MCF and, although virus DNA was not detected, had a rising AlHV-1 −specific antibody titre in the blood after challenge and was therefore classified as CD III.

In Group 3, two animals survived with no clinical signs and no detection of virus DNA post-challenge and were classified as CD I. Six animals succumbed to MCF with clinical signs and virus DNA detected post-challenge and were classified as CD II.

In Group 4, four animals succumbed to MCF with clinical signs and virus DNA detected post-challenge and were classified as CD II, while two animals survived with no clinical signs and no detection of virus DNA in the blood post-challenge. These animals also had no AlHV-1 − specific antibody response and were classified as CD I. A further two animals (numbers 914 & 937) survived but had clinical signs indicative of MCF. Both developed AlHV-1 − specific antibody responses after challenge but only one had detectable virus DNA in the blood post-challenge. These were classified as CD III.

In Group 5, six animals succumbed to MCF with clinical signs and virus DNA detected post-challenge and were classified as CD II. The remaining two animals (numbers 919 & 921) showed clinical signs of MCF but neither had virus DNA in the blood post-challenge nor an AlHV-1 − specific antibody response. One animal died and the other survived. These were classified as CD IV (possible AlHV-1 infection). The numbers of each case type per group are summarized in Table 3.

### 3.4 Comparison of groups

When infection status (‘uninfected’ versus ‘infected’) of Groups 1 and 2 were compared to their respective adjuvant-only control Groups 4 and 5, the difference in the total number of infected versus uninfected cases was not significant (*p* = 0.13 and 0.2 respectively). When infection status of Groups 1 & 2 combined were compared to unvaccinated cattle Groups 4 & 5 combined the difference in the number of infected cattle was just outside the conventional threshold of significance (*p* = 0.06). Cattle classified as ‘possibly infected’ were not included in this analysis. The results were not substantively different when cattle with evidence of prior exposure to AlHV-1 were removed. FliC appeared to have a negative effect on post-challenge survival (comparing Groups 1 and 3) but this effect was not significant (*p* = 0.06).

### 3.5 The relationship between serological response and protection

**Fig. 4:**
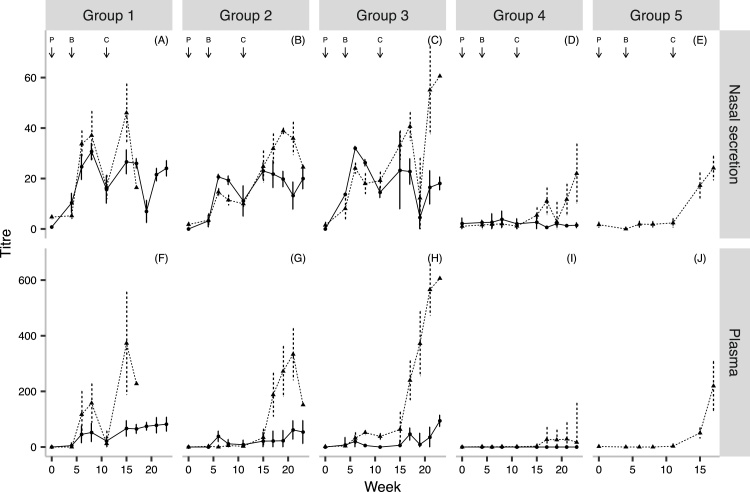
AlHV-1 – specific antibody titres and outcome: The geometric mean nasal secretion (plots A–E) and plasma (plots F–J) AlHV-1 – specific antibody titres were plotted for each treatment group according to the outcome following challenge (uninfected = solid line; infected = dashed line; P = primary vaccination, B = booster and C = challenge). Group 1, atAlHV-1 + Emulsigen^®^; Group 2, atAlHV-1 + FliC; Group 3, atAlHV-1 + Emulsigen^®^ + FliC; Group 4, Emulsigen^®^; Group 5, FliC.

We calculated the geometric mean nasal secretion and plasma AlHV-1-specific antibody titres for ‘infected’ and ‘uninfected’ cattle in each group for each time point. The results are illustrated in Fig. 4. In the pre-challenge titres of infected and uninfected cattle there were no significant differences between any of the groups. This was also the case with the pre-challenge AlHV-1-neutralizing antibody titres. In contrast, in Groups 1, 2 and 3, the post-challenge plasma and nasal secretion titres of ‘infected’ cattle were all significantly higher than ‘uninfected’ cattle (*p* *<* 0.04).

### 3.6 The relationship between prior exposure and survival in control group cattle

Of the sixteen control group cattle, four had evidence of prior exposure to AlHV-1 through baseline seropositivity (numbers 901 & 926) or evidence of AlHV-1 DNA in blood mononuclear cells (numbers 914 & 937). Of these cattle, three survived (numbers 914, 926 & 937) (Table 2). A comparison of the proportions of pre-exposed and non pre-exposed control group cattle that survived and died was just outside the conventional level of significance (odds ratio = 11.8; *p* = 0.06).

### 3.7 Vaccine efficacy and comparisons with previous trials

An efficacy calculation (for preventing infection) was performed for the formulations used in this trial and the field, Russell and Haig trials. The results, which are shown in Supporting information 2, indicate that the calculated efficacies in the UK trials (80–90%) were higher than the Tanzanian trials (50–60%), however the wide confidence intervals indicate that these differences were not significant. The calculated efficacy of the atAlHV-1 + Emulsigen^®^ vaccine formulation following experimental virus challenge was 50% in this trial in which SZC cattle were used whilst it was 81.5% in the Russell trial in which FH were used.

## 4 Discussion

This was the first experimental trial to investigate the efficacy of a new immunization strategy against MCF in an East African breed of cattle. The vaccine was developed and tested in the UK using FH cattle and, using the same experimental design (Haig et al., 2008, Russell et al., 2012), this study was carried out to test its efficacy in SZC under local management conditions. In addition, we investigated whether the adjuvant FliC improves the protective immune response.

All tested immunization regimes (Groups 1–3) stimulated seroconversion in all SZC cattle, generating mucosal (nasal secretion) and systemic (plasma) AlHV-1-specific and neutralizing antibodies. However, comparing immune responses in Groups 1 − 3, it is clear that the inclusion of Emulsigen^®^ was crucial to the induction of high titre antibody responses and that the use of FliC did not enhance this response. Despite the 100% seroconversion in vaccinated cattle, when outcomes were compared between vaccinated and control groups, the differences were not significant. Nonetheless Group 1 had the highest survival rate (75%), the smallest number of animals that became infected, and the highest antibody titres. This suggests that, in SZC cattle, the atAlHV-1 + Emulsigen^®^ mixture was the most effective of the tested formulations.

The pre-challenge antibody titres did not appear to impact whether an animal became infected or not. This was surprising and suggests that there are other immunological and/or physiological factors involved in determining whether an animal becomes infected. These might include the effective challenge dose delivered to the correct site of infection; the specific antigens or epitopes recognized in protected versus infected cattle; and the presence of additional subclinical infections at the time of challenge.

The post-challenge antibody titres were significantly higher in the 14 cattle in Groups 1–3 that became infected than those that did not. This boosted (anamnestic) response in the infected cattle indicates that the virus managed to infect these animals, stimulating memory cells to produce more antibodies. This was consistent with previous work (Parameswaran et al., 2014, Russell et al., 2012). As 12 of these cattle died, we conclude that, if the vaccine fails to protect against infection, any antibody response that takes place after infection is not protective.

The comparison of vaccine efficacies between the field trial (56%) and this trial (50%) suggests that the mode of challenge did not greatly affect the efficacy of the vaccine, even though direct intra-nasal administration of a high virus dose is likely to represent a more severe challenge than most field exposures. Despite no FH cattle being used in this trial, which makes direct comparison difficult, the comparison of vaccine efficacies across this study and the Russell trial allows us to speculate whether the sub-species of cattle used (*Bos taurus* (FH) or *B. indicus* (SZC)) might impact efficacy. Indeed, the calculated efficacy was 31.5% less when the vaccine was used in SZC. Vaccine efficacy will decrease following a reduction in either the number of protected vaccinated animals or the number of cases in unvaccinated animals. Notably the risk of vaccinated SZC cattle becoming infected (38%) was more than double that of FH (17%). This could suggest that the vaccine is not as effective in SZC at stimulating the appropriate immune response to protect against AlHV-1 infection. Conversely the risk of unvaccinated SZC cattle becoming infected following experimental viral challenge (75%) was considerably lower than for FH cattle (90%). It is possible that differences in animal husbandry and the treatments given to the SZC cattle may have contributed to a reduction in the effectiveness of the viral challenge. It is also possible that, following repeated annual exposure to migrating wildebeest, the African breed is more resistant to AlHV-1. This view is supported by recent genetic studies on the closely-related East African Shorthorn Zebu, which provide evidence of selection for traits related to survival in the African environment, including factors such as resistance to endemic pathogens (Bahbahani et al., 2015, Murray et al., 2013). Additionally the increased survival of SZC seen could be the result of natural adaptive immunity following previous exposure. Indeed, in this trial, post-hoc analysis of baseline samples collected before challenge indicated that three of the five surviving control group cattle had evidence of a prior exposure to AlHV-1. Thus previous exposure to MCF virus might have influenced the outcome of subsequent infection. This remains to be addressed experimentally. It is possible that the two animals that were sero-positive in the pre-trial sample could simply be the result of infection with viruses that cross-react with the ELISA used here. While it is likely that other MCF virus infections would induce cross-reactive antibodies detectable by AlHV-1 ELISA, it has also been reported that bovine herpesvirus-4 (BoHV-4) antibodies can detect AlHV-1 infected cells by immunofluorescence (Dewals et al., 2005). However previous testing did not show cross-reactivity with BoHV-4 specific sera in the direct MCF ELISA (Fraser et al., 2006).

The unexpected survival of control group cattle could also be related to the effects of the ECF vaccination administered to all cattle before the trial. Given that both MCF and ECF are associated with the proliferation of T-cells (Dewals et al., 2008, Kessy and Matovelo, 2009, Thonur et al., 2006) any non-specific suppression of T-cell proliferation as a consequence of ECF vaccination could provide some protection from MCF pathogenesis. This hypothesis will be investigated in a subsequent field trial.

The vaccine’s efficacy determined by this trial and the field trial is estimated to be 50 − 56%. Although likely to be too low for annual vaccination of cattle to replace the traditional MCF avoidance strategy, a partially protective MCF vaccine could provide protection to valuable cattle that, because of changes in land-use, cannot be moved away from oncoming wildebeest. Furthermore, given there are very few, if any, herpesvirus vaccines that effectively prevent infection and the establishment of latency, we consider the proportion of SZC vaccinated animals that did not become infected in the face of intense challenge in this and the field trial as promising for future vaccine improvement strategies.

We also assessed FliC as an adjuvant. The *in-vitro* analysis showed that FliC stimulation of bovine TLR5 induced a significant CXCL-8 response in HEK cells, although this was lower than that induced via human TLR5. The addition of FliC to the vaccine formulation (Groups 2 & 3) reduced antibody titres and survival when compared with Group 1, although this latter effect was just outside the conventional levels of significance (*p* = 0.06). These data suggest that FliC is unlikely to enhance protection against MCF.

WA-MCF has a case-fatality ratio greater than 96% (Plowright et al., 1960). The finding that 15% of the trial cattle had evidence of prior AlHV-1 infection was therefore surprising. Non-fatal infections have been reported in SA-MCF (Moore et al., 2010, Otter et al., 2002) and serological evidence of non-fatal infections was described in the field trial (Lankester et al., 2016). These findings add further evidence that non-fatal outcomes are a feature of WA-MCF and that the case-fatality ratio could be lower than previously described.

The cell biology and pathogenesis of MCF are poorly understood. The fact that four cattle were PCR positive at baseline suggests that, following initial infection, virus was not eliminated from cattle that survived the infection. It is not clear whether the virus became latent, residing in certain body tissues as it does in the carrier host, nor whether it might cause MCF at a later stage.

In summary, immunization with atAlHV-1 induces an oro-nasopharyngeal antibody response in FH and SZC and there is evidence that, when combined with Emulsigen^®^, the vaccine mixture induces a partial protective immunity in SZC. A larger study is required to better quantify this effect. We have shown that direct challenge with the pathogenic AlHV-1 virus is effective at inducing MCF in SZC. We have also provided evidence that the atAlHV-1 + Emulsigen^®^ formulation may be less effective at stimulating a protective immune response in SZC cattle than FH cattle. Furthermore, and in support of the field trial, we have provided evidence that non-fatal AlHV-1 infections are relatively common and we speculate that there could be resistance to fatal MCF in SZC cattle, possibly through genetic background, previous (sub-clinical) exposure to AlHV-1 or alternative acquisition of a level of inherent immunity. Finally, we demonstrated that FliC is not an appropriate adjuvant for the atAlHV-1 vaccine.

## Figures and Tables

**Table 1:** Immunization treatment groups and inoculations.

Group (*n*)	Primary/Boost immunization (week 0/4)^a^^,^^b^	Virus challenge (week 10)^c^^,^^d^
1 (8)	10^7^ TCID_50_ atAlHV-1 in 20% v/v Emulsigen^®^	10^4^ TCID_50_ AlHV-1
2 (8)	10^7^ TCID_50_ atAlHV-1 in 1 mg FliC	10^4^ TCID_50_ AlHV-1
3 (8)	10^7^ TCID_50_ atAlHV-1 in 20% v/v Emulsigen^®^ + FliC	10^4^ TCID_50_ AlHV-1
4 (8)	20% v/v Emulsigen^®^ alone	10^4^ TCID_50_ AlHV-1
5 (8)	1 mg FliC alone	10^4^ TCID_50_ AlHV-1

a at AlHV-1 = attenuated AlHV-1 virus, FliC = flagellin monomer.

b Prime and boost inoculations for each group were identical in composition, given as a 1 ml injection intramuscularly to the upper neck region.

c TCID_50_ = 50% tissue-culture-infectious dose.

d Pathogenic AlHV-1 challenge was given as a 10 ml dose inoculated intranasally.

**Table 2:** Summary of the outcomes of the trial.

Group: vaccination	Cattle ID	Baseline^a^ PCR/Serology (titre)	Survived/Died (days post challenge)^b^	Clinical signs	PCR	Antibody^c^	Case Definition^d^	Infection status^e^
1: atAlHV-1 + Em	903		Survived	Healthy	Neg	reducing	I	Uninfected
	912		Survived	Healthy	Neg	level	I	Uninfected
	913		Survived	Healthy	Neg	rising	III	Infected
	918		Survived	Healthy	Neg	level	I	Uninfected
	923		Died (22)	Sick	Pos	rising	II	Infected
	927		Survived	Healthy	Neg	level	I	Uninfected
	928	Pos	Survived	Healthy	Neg	level	I	Uninfected
	930		Died (38)	Sick	Pos	rising	II	Infected

2: atAlHV-1 + FliC	906		Survived	Healthy	Neg	level	I	Uninfected
	908		Died (35)	Sick	Pos	rising	II	Infected
	915		Survived	Healthy	Neg	level	I	Uninfected
	925		Died (66)	Sick	Pos	rising	II	Infected
	929		Died (38)	Sick	Pos	rising	II	Infected
	936	Pos	Died (56)	Sick	Pos	rising	II	Infected
	938		Survived	Healthy	Neg	level	I	Uninfected
	940		Survived	Sick	Neg	rising	III	Infected

3: atAlHV-1 +Em + FliC	905		Died (31)	Sick	Pos	rising	II	Infected
	907		Died (59)	Sick	Pos	rising	II	Infected
	910		Survived	Healthy	Neg	level	I	Uninfected
	916		Died (50)	Sick	Pos	rising	II	Infected
	920		Died (38)	Sick	Pos	rising	II	Infected
	922		Died (38)	Sick	Pos	rising	II	Infected
	924		Died (88)	Sick	Pos	rising	II	Infected
	935		Survived	Healthy	Neg	level	I	Uninfected

4: Em only control	904		Died (59)	Sick	Pos	sero-pos	II	Infected
	909		Survived	Healthy	Neg	no titre	I	Uninfected
	914	Pos	Survived	Sick	Pos	sero-pos	III	Infected
	917		Died (38)	Sick	Pos	sero-pos	II	Infected
	926	28	Survived	Healthy	Neg	no titre	I	Uninfected
	931		Died (35)	Sick	Pos	sero-pos	II	Infected
	933		Died (35)	Sick	Pos	sero-pos	II	Infected
	937	Pos	Survived	Sick	Neg	sero-pos	III	Infected

5: FliC only control	901	35	Died (28)	Sick	Pos	sero-pos	II	Infected
	902		Died (22)	Sick	Pos	sero-pos	II	Infected
	911		Died (31)	Sick	Pos	sero-pos	II	Infected
	919		Survived	Sick	Neg	no titre	IV	Possibly infected
	921		Died (26)	Sick	Neg	no titre	IV	Possibly infected
	932		Died (35)	Sick	Pos	sero-pos	II	Infected
	934		Died (38)	Sick	Pos	sero-pos	II	Infected
	939		Died (38)	Sick	Pos	sero-pos	II	Infected

a Baseline serology/PCR: animals with AlHV-1-specific antibodies at day 0 of the experiment (pre-vaccination) are shown by the measured ELISA titre value (cut off value of 20), while animals that had detectable AlHV-1 DNA are indicated as Pos.

b Died: Whether an animal survived or died is indicated, with the number of days that an animal died post-challenge given in parentheses.

c Antibody: summary of plasma ELISA titres post-challenge. For vaccinated animals (Groups 1, 2, 3), ‘rising’ titre after challenge indicates infection with AlHV-1, while for control animals (Groups 4 and 5), presence of AlHV-1-specific antibodies (‘sero-pos’) indicates infection with AlHV-1.

d Case definition: Cases (as described in the text) are defined as I (not infected), II (fatal AlHV-1 infection), III (non-fatal AlHV-1 infection) and IV (possible AlHV-1 infection).

e Infection status: indicated as ‘infected’ for case definitions (CD) II (fatal AlHV-1 infection) and III (non-fatal AlHV-1 infection); or ‘uninfected’ for cases with CD I (not infected). Cases classed as CD IV (possible AlHV-1 infection) are indicated as ‘possibly infected’. (atAlHV-1 = attenuated AlHV-1 virus, Em = Emulsigen^®^, FliC = bacterial flagellin monomer).

**Table 3:** Summary of case outcomes:.

Group^a^	Died^b^	Survived^b^	MCF cases^c^
1 (atAlHV-+ Em)	●●	●○○○○○	3
2 (atAlHV-+ FliC)	●●●●	●○○○	5
3 (atAlHV-+ Em +FliC)	●●●●●●	○○	6
4 (Em)	●●●●	●●○○	6
5 (FliC)	●●●●●●□	□	6

a atAlHV-1 = attenuated AlHV-1 virus, Em = Emulsigen^®^, FliC = flagellin monomer.

b ○ Uninfected: animals with no evidence of MCF infection (CD I). ● Infected: animals with evidence of infection through PCR or virus-specific antibody response: CD II, died; CD III, survived. □ Possibly infected: Animals with MCF signs only (CD IV).

c MCF cases (●) equals CD II (Fatal AlHV-1 infection) plus III (Non-fatal AlHV-1 infection).
