# Supplementary material for: The efficacy of alcelaphine herpesvirus-1 (AlHV-1) immunization with the adjuvants Emulsigen® and the monomeric TLR5 ligand FliC in zebu cattle against AlHV-1 malignant catarrhal fever induced by experimental virus challenge
Source: Vet Microbiol. 2016 Nov 15;195:144–53. doi: 10.1016/j.vetmic.2016.09.019 (PMC5081063; doi:10.1016/j.vetmic.2016.09.019)
Supplement: Supplementary file 1 [file mmc1.pdf]

Supporting information 1:

Group / vaccination	Cattle ID	Day zero Baseline	Day 105	Day 133	Day 163	Terminal sample (days)
1: atAlHV-1 +Emulsigen	903	Neg	Neg	Neg	Neg	Sur
	912	Neg	Neg	Neg	Neg	Sur
	913	Neg	Neg	Neg	Neg	Sur
	918	Neg	Neg	Neg	Neg	Sur
	923	Neg				Pos (99)
	927	Neg	Neg	Neg	Neg	Sur
	928	Pos	Neg	Neg	Neg	Sur
	930	Neg	Neg			Pos (115)
2: atAlHV-1+FliC	906	Neg	Neg	Neg	Neg	Sur
	908	Neg	Pos			Pos (112)
	915	Neg	Neg	Neg	Neg	Sur
	925	Neg	Neg	Neg	Pos	Pos (143)
	929	Neg	Pos			Pos (115)
	936	Pos	Neg			Pos (133)
	938	Neg	Neg	Neg	Neg	Sur
	940	Neg	Neg	Neg	Neg	Sur
3: atAlHV-1 +Em+FliC	905	Neg	Pos			Pos (108)
	907	Neg	Neg	Neg	Pos	Pos (146)
	910	Neg	Neg	Neg	Neg	Sur
	916	Neg	Pos			Pos (127)
	920	Neg	Pos			Pos (115)
	922	Neg	Pos			Pos (115)
	924	Neg	Neg	Neg	Pos	Pos (165)
	935	Neg	Neg	Neg	Neg	Sur
4: Em only control	904	Neg	Neg	Pos		Pos (136)
	909	Neg	Neg	Neg	Neg	Sur
	914	Pos	Pos	Pos	Neg	Sur
	917	Neg	Pos			Pos (115)
	926	Neg	Neg	Neg	Neg	Sur
	931	Neg	Pos			Pos (112)
	933	Neg	Pos			Pos (112)
	937	Pos	Neg	Neg	Neg	Sur
5: FliC only control	901	Neg	Pos			Pos (105)
	902	Neg				Pos (99)
	911	Neg	Pos			Pos (108)
	919	Neg	Neg	Neg	Neg	Sur
	921	Neg				Neg (103)
	932	Neg	Pos			Pos (112)
	934	Neg	Pos			Pos (115)
	939	Neg	Pos			Pos (115)

Samples collected at the start of the trial (baseline day zero) and at three time points thereafter are shown. The number of days to death and the result of the PCR assay performed on blood samples collected at this time ('Terminal sample') are shown in the far right hand column (Sur = animal survived, Pos = Positive, Neg = Negative).
