# Supplementary material for: The efficacy of alcelaphine herpesvirus-1 (AlHV-1) immunization with the adjuvants Emulsigen® and the monomeric TLR5 ligand FliC in zebu cattle against AlHV-1 malignant catarrhal fever induced by experimental virus challenge
Source: Vet Microbiol. 2016 Nov 15;195:144–53. doi: 10.1016/j.vetmic.2016.09.019 (PMC5081063; doi:10.1016/j.vetmic.2016.09.019)
Supplement: Supplementary file 2 [file mmc2.pdf]

Supporting information 2:

Trial	Group	No. infected	No. not infected	Risk	Vaccine efficacy (95% CIs)
Formula	Vacc	a	b	$Rv = \frac{a}{a+b}$	$VE = \left(1 - \frac{Rv}{Ru}\right) \times 100$
	Unvacc	c	d	$Ru = \frac{c}{c+d}$	
Haig	Vacc	1	9	0.10	90% (35.8 – 98.4%)
	Unvacc	6	0	1.00	
Russell	Vacc	3	15	0.17	81.5% (46.9 – 93.5%)
	Unvacc	9	1	0.90	
Field	Vacc	19	76	0.20	56% (29.5 – 72%)
	Unvacc	41	51	0.45	
Tz challenge	Vacc	3	5	0.38	50% (-33.2 – 81.2%)
	Unvacc	6	2	0.75	

Vaccine efficacy (VE) formula and calculations: In the VE formula *Rv* and *Ru* are the risk of becoming infected in vaccinated and unvaccinated cattle respectively. Data from four vaccine trials testing the attenuated AlHV-1 vaccine are also shown (Haig = Haig et al., 2008; Russell = Russell et al., 2012; Field = Lankester et al., 2016; Tz challenge = this trial). The number of vaccinated (Vacc) and unvaccinated (Unvacc) animals that became infected, the calculated risk of becoming infected in each group (Risk) and the resultant vaccine efficacies (and 95% confidence intervals) are shown.
